# Assessing the mitochondrial safety profile of the molnupiravir active metabolite, β-d-N4-hydroxycytidine (NHC), in the physiologically relevant HepaRG model

**DOI:** 10.1093/toxres/tfae012

**Published:** 2024-02-07

**Authors:** Robyn T Kiy, Saye H Khoo, Amy E Chadwick

**Affiliations:** Department of Pharmacology and Therapeutics, University of Liverpool, Sherrington Building, Ashton Street, Liverpool, L69 3GE, United Kingdom; Department of Pharmacology and Therapeutics, University of Liverpool, Sherrington Building, Ashton Street, Liverpool, L69 3GE, United Kingdom; Tropical Infectious Diseases Unit, Royal Liverpool University Hospital, Prescot Street, Liverpool, L7 8XP, United Kingdom; Department of Pharmacology and Therapeutics, University of Liverpool, Sherrington Building, Ashton Street, Liverpool, L69 3GE, United Kingdom

**Keywords:** β-d-N4-hydroxycytidine (NHC), molnupiravir, HepaRG, mitochondrial dysfunction, nucleoside analogue, drug-induced mitochondrial toxicity

## Abstract

**Background:**

β-d-N4-Hydroxycytidine (NHC) is the active metabolite of molnupiravir, a broad-spectrum antiviral approved by the MHRA for COVID-19 treatment. NHC induces lethal mutagenesis of the SARS-CoV-2 virus, undergoing incorporation into the viral genome and arresting viral replication. It has previously been reported that several nucleoside analogues elicit off-target inhibition of mitochondrial DNA (mtDNA) or RNA replication. Although NHC does not exert these effects in HepG2 cells, HepaRG are proven to be advantageous over HepG2 for modelling nucleoside analogue-induced mitochondrial dysfunction. Therefore, the objective of this work was to assess the mitotoxic potential of NHC in HepaRG cells, a model more closely resembling physiological human liver.

**Methods:**

Differentiated HepaRG cells were exposed to 1–60 μM NHC for 3–14 days to investigate effects of sub-, supra-, and clinically-relevant exposures (in the UK, molnupiravir for COVID-19 is indicated for 5 days and reported Cmax is 16 μM). Following drug incubation, cell viability, mtDNA copy number, mitochondrial protein expression, and mitochondrial respiration were assessed.

**Results:**

NHC induced minor decreases in cell viability at clinically relevant exposures, but did not decrease mitochondrial protein expression. The effects on mtDNA were variable, but typically copy number was increased. At supra-clinical concentrations (60 μM), NHC reduced mitochondrial respiration, but did not appear to induce direct electron transport chain dysfunction.

**Conclusions:**

Overall, NHC does not cause direct mitochondrial toxicity in HepaRG cells at clinically relevant concentrations, but may induce minor cellular perturbations. As HepaRG cells have increased physiological relevance, these findings provide additional assurance of the mitochondrial safety profile of NHC.

## Introduction

Since the emergence of severe acute respiratory syndrome coronavirus 2 (SARS-CoV-2) in December 2019, it has remained a global priority to develop and implement effective therapeutics for the prevention or treatment of COVID-19.^1^ At the time of writing, over 3,300 interventional clinical trials are recruiting, ongoing, or completed worldwide.^2^ As throughout the pandemic, the majority of these trials are investigating the efficacy of drug(s) which are approved by regulators for indications other that the treatment of COVID-19.^2^^,^^3^ However, there has also been a push to test and market novel therapeutic compounds, of which molnupiravir is one.

Molnupiravir is a broad-spectrum antiviral which was approved by the UK Medicines and Healthcare products Regulatory Agency (MHRA) in 2021, and authorized for emergency use by the U.S. FDA, for the treatment of mild to moderate coronavirus disease symptoms in adults who have tested positive for SARS-CoV-2.^4^ The 5′-isopropyl ester prodrug of the ribonucleoside analogue β-d-N^4^-Hydroxycytidine (NHC), molnupiravir is reported to be over 100-fold more active than ribavirin or favipiravir against SARS-CoV-2 infection.^5^ Originally developed by the Emory Institute of Drug Development in 2013 as part of the search for an orally available antiviral effective against Venezuelan equine encephalitis virus (VEEV), NHC was subsequently shown to effectively inhibit replication of the SARS-CoV-2 virus in vitro and in animal models.^6–9^

NHC works by inducing lethal mutagenesis, and once phosphorylated to the pharmacologically active 5′-triphosphate form is incorporated by the SARS-CoV-2 RNA-dependent RNA polymerase (RdRp) into the resulting RNA in place of cytidine triphosphate (CTP) or uridine triphosphate (UTP).^5^^,^^9–11^ When using the resultant RNA as a template, NHC induces RNA mutagenesis by the misincorporation of G or A nucleotides.^10^ NHC exhibits activity across a broad spectrum, and has also been shown to arrest replication of SARS-CoV, MERS-CoV, and influenza viruses.^6^^,^^7^ However, uncertainty remains regarding the potential of NHC to cause mutagenesis of host mitochondrial RNA (mtRNA) and mtRNA products, a phenomenon which has been induced by several nucleoside analogues in the clinic via multiple mechanisms targeting the synthesis of mitochondrial DNA, RNA, or RNA products.^5^^,^^12–14^

Recent research has shown that neither NHC or its parent compound molnupiravir elicit significant mitochondrial dysfunction In HepG2 cells following 48 h exposure to sub-toxic concentrations.^15^ However, certain features of HepG2 cells, including their rapid proliferation rate and subsequent constrained viable drug exposure window, mean that they are not always the most appropriate model for recapitulating drug-induced mitochondrial dysfunction in vitro*.* One relevant example of this regards fialuridine, a nucleoside analogue which caused severe multisystem toxicity during a clinical trial in 1993, resulting in the death of five patients.^16^ The toxicity of fialuridine has since been well characterized, and it remains an important example of a compound for which the primary mechanism of hepatotoxicity is mitochondrial toxicity.^17–19^ However, when investigating fialuridine toxicity in vitro, the relatively short potential dosing period of HepG2 cells (approximately 72 h, compared to 28 days for differentiated HepaRG cells) is a substantial limitation meaning valuable clinically-relevant mechanistic insight is missed.^20^ The use of the hepatic progenitor cell line HepaRG by Jolly et. al enabled this barrier to be overcome, ultimately revealing valuable mechanistic insight into fialuridine-induced mitotoxicity. Clinically, molnupiravir indicated for COVID-19 is administered for five days, longer than it would be practically possible to dose two dimensional cultures of HepG2 cells.^21^ To recapitulate the clinically-relevant dosing period in an in vitro model with characteristics resembling those of primary human hepatocytes (PHH), we explored whether NHC induces perturbations in mitochondrial function following extended dosing periods in HepaRG cells. We also explored additional exposure periods beyond five days (up to 14 days) in order to investigate the longer-term effects. As the COVID-19 landscape continues to constantly evolve, the potential requirement to update pharmaceutical response is undeniable; it is beneficial to know as much about these therapies as possible.

## Materials and methods

### Materials

HepaRG cells were purchased from Biopredic International (Saint Grégoire, France). QIAamp DNA Mini Kits were purchased from Qiagen (Manchester, UK). Nunc flasks, 6-well plates, enhanced chemiluminescence (ECL) substrate, Tween™ 20, glucose, glycine, tris base, methanol, sodium chloride, potassium chloride, hydrochloric acid, and dimethyl sulfoxide (DMSO) were purchased from Thermo Fisher Scientific (Loughborough, UK). Tissue culture-treated 96-well plates were purchased from STARLAB (Milton Keynes, UK). White and clear 96-well microplates were purchased from Greiner Bio-One (Gloucestershire, UK). All Extracellular Flux Analyser (XFe96) consumables were purchased from Agilent (Santa Clara, USA). Nitrocellulose membrane was purchased from GE Healthcare (Buckinghamshire, UK). Hyperfilm™ ECL™ was purchased from Cytiva (Buckinghamshire, UK). Blotting-grade blocker and Precision Plus Protein™ Kaleidoscope™ Prestained Protein Standards were purchased from Bio-Rad (California, USA). Total OXPHOS Human WB antibody was purchased from Abcam (Cambridge, UK), and GAPDH antibody was purchased from Proteintech (Manchester, UK). Williams’ Medium E, penicillin-streptomycin, insulin, hydrocortisone, sodium pyruvate, sodium bicarbonate, galactose, L-glutamine, sucrose, bicinchoninic acid (BCA) kit, all ATP assay reagents, and all mitochondrial stress test compounds were purchased from Sigma Aldrich (Dorset, UK). NHC was obtained from Toronto Research Chemicals (Toronto, Canada). All other materials were purchased from Life Technologies (Paisley, UK).

### Methods

#### HepaRG cell culture

Undifferentiated HepaRG cells were thawed and grown (37 °C in 5% CO_2_) for two weeks in HepaRG growth medium consisting of William’s E medium supplemented with 10% (v/v) FBS, 1% (v/v) penicillin-streptomycin, L-glutamine (2 mM), 5 ug/mL insulin, and hydrocortisone (50 μM), with twice-weekly media changes. Cells in HepaRG growth medium were then seeded on to collagen-coated plates at densities as per supplier’s guidance. After a 2-week growth period, cells were maintained with HepaRG differentiation medium (HepaRG growth medium supplemented with 1.7% (v/v) DMSO) for 2 weeks, again with twice-weekly media changes. During this period, the bipotent progenitor cell line HepaRG cells undergo a differentiation to a stable monolayer of morphologically-distinct hepatocyte-like and biliary-like cells.^22^^,^^23^ Once fully differentiated, HepaRG cells can be maintained in differentiation medium to allow them to be used experimentally for a further 28 days. HepaRG cells were supplied at passage 12 and used between passages 15–20 in order to minimize risk of genetic instability and deviation from normal phenotype that can occur as a result of excessive passaging.

#### NHC dosing protocol

Drug treatment of HepaRG cells began once they had undergone a full differentiation. Concentrations of NHC were chosen to encompass subclinical, clinically relevant, and supra-clinical levels based off the Cmax of 4,180 ng/mL (16.13 μM) reported by Khoo et al. following five days of molnupiravir treatment.^24^ NHC 200× stock solutions were prepared in DMSO, and final concentrations ranging from 1–60 μM were replaced in fresh HepaRG growth medium twice per week. The final concentration of DMSO was maintained at a constant 0.5% (v/v) across all treatments, and a vehicle control was included within each experiment. HepaRG cells were treated for 3–14 days either continuously, or with a “recovery period” during which cells were maintained in HepaRG differentiation medium (Table 1), intended to capture the potential delay in onset of mitochondrial toxicity following cessation of treatment with nucleoside analogues.^25^

**Table 1:** NHC dosing regimen for HepaRG cells.

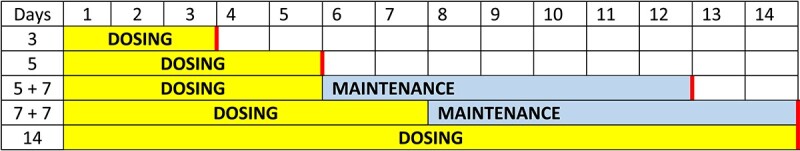

Dosing periods are shown in yellow and indicated by the word “DOSING”, and post-treatment recovery periods in differentiation medium are shown in blue and indicated by the word “MAINTENANCE”. Bold red lines indicate timepoints at which experiments were conducted.

HepaRG cells were also exposed to the known mitotoxic drug chloramphenicol^26–28^ for up to 28 days in order to provide a positive control for drug-induced mitochondrial dysfunction (Supplementary Data). Chloramphenicol induces mitochondrial dysfunction via the inhibition of mitochondrial protein synthesis. Despite inducing mitochondrial dysfunction via a different mechanism to molnupiravir, chloramphenicol was selected as an appropriate positive control as treatment with this compound resulted in mitochondrial dysfunction in HepaRG cells which was able to be detected across all included endpoints.

#### Assessment of cellular ATP content

ATP assays were conducted to assess cell viability following treatment with NHC. Following the respective drug incubation periods, media was removed from the cells and they were lysed in somatic cell ATP releasing reagent (50 μL/well). The lysate was used to determine the ATP and protein content of each well using the Sigma adenosine 5′-triphosphate bioluminescent somatic cell assay kit and Pierce™ BCA protein assay kit respectively according to manufacturer’s recommendations. ATP content was then normalized to protein content for each corresponding well. Total ATP levels are reported as percentage of the vehicle, and protein content is reported as μg/well.

#### Quantification of cellular nuclear and mitochondrial DNA content using qPCR

Following drug incubation, DNA purification was performed using the QIAamp DNA Mini Kit according to the manufacturer’s guidelines. Briefly, HepaRG cells were detached using a cell scraper and centrifuged at 300 × *g* for 5 min. Cells were resuspended in 200 μL 1× PBS and 20 μL of proteinase K was added, followed by 200 μL Buffer AL. After vortexing, samples were incubated for 10 min at 56 °C before the addition of 200 μL absolute ethanol. This mixture was then applied to QIAamp Mini spin columns and centrifuged at 6,000 × *g* for 1 min. The spin column was then moved to a new collection tube, where 500 μL of Buffer AW1 was added before centrifuging at 6,000 × *g* for 1 min. An equal volume of Buffer AW2 was then added and samples were centrifuged at 20,000 × *g* for 4 min. Finally, QIAamp Mini spin columns were placed into clean collection tubes and incubated with 200 μL Buffer AE for 5 min before a final centrifugation step of 1 min at 6,000 × *g.* Total gDNA concentration was measured using a NanoDrop® ND-1000 UV-Vis Spectrophotometer.

Relative mitochondrial DNA (mtDNA) copy number was determined by real-time pre-designed PCR TaqMan™ detection kits designed to target the nuclear housekeeping gene RNaseP and the mitochondrial MT-ND1 gene using TaqMan™ genotyping Master Mix (Table 2). PCR reactions were performed according to standard TaqMan™ conditions according to the manufacturer (95 °C for 10 min, followed by 40 cycles of 95 °C for 15 s, 60 °C for 1 min). PCR assays were performed in triplicate for each DNA sample. Levels of mitochondrial ND-1 were subsequently normalized to nuclear RNaseP and treated samples were normalized to the respective vehicle control.

**Table 2:** Details of the nDNA and mtDNA TaqMan™ primers used for qPCR.

Gene	Probe	Location
RNase P (nDNA)	VIC dye-labelled TAMRA probe	Chromosome 14, cytoband 14q11.2
ND-1 (mtDNA)	FAM dye-labelled MGB probe	mtDNA 3,307–4,262

#### Detection and relative quantification of mitochondrial/nuclear DNA-encoded mitochondrial proteins

Cellular lysates were used to prepare samples containing 20 μg protein for loading in LDS sample buffer. Samples were heat denatured for 30 min at 37 °C then resolved according to molecular weight in NuPAGE® 4–12% Bis-Tris pre-cast gels. Following electrophoresis, samples were transferred to nitrocellulose membranes then blocked with 10% (w/v) non-fat dry milk (NFDM) in Tris-buffered saline with 1 (v/v) Tween 20 (TBS-T). Blots were then probed with primary antibodies (Table 3) and then washed and probed with HRP-conjugated secondary antibodies diluted 1:10,000 in 2% (w/v) NFDM in TBS-T. Protein bands were visualized using an ECL system and captured using X-ray film, and subsequent densitometry analysis was conducted using ImageJ 1.48 software.

**Table 3:** Western blot incubation conditions for primary and secondary antibodies.

Protein(s) of interest	Antibody product code	Molecular weight (kDa)	1° antibody dilution	1° antibody % milk	HRP-conjugated secondary antibody
ATP5A, UQCRC2, SDHB, COX II, NDUFB8	ab110411	54, 48, 29, 22, 18	1:1000	10	Anti-mouse
GAPDH	60,004–1-Ig	36	1:5000	5	Anti-mouse

Summary of primary antibodies, dilution conditions and appropriate secondary HRP-conjugated secondary antibody.

#### Assessment of mitochondrial function using Agilent Seahorse technology

On the day of the assay, cells were washed twice with seahorse media (Seahorse XF DMEM Medium (pH 7.4) supplemented with 2 mM L-glutamine, 1 mM sodium pyruvate, and 25 mM glucose), before 175 μL fresh seahorse media was added and cells were equilibrated in a non-CO_2_ incubator at 37 °C for one hour before beginning the assay. The Seahorse mitochondrial stress test assay was used, consisting of consecutive additions of oligomycin (1 μM), FCCP (0.25 μM), and rotenone/antimycin A (1 μM) (concentrations chosen following optimization assays). Rates of oxygen consumption and extracellular acidification were recorded at regular intervals throughout the assay. Parameter calculations were performed using the Seahorse XF Cell Mito Stress Test Report Generator.

### Statistical analyses

All assays were conducted with at least three biological replicates. Data were analysed by Analysis of Variance followed by Tukey or Dunnett’s multiple comparisons test using GraphPad Prism 9 (Boston, MA, USA).

## Results

### An extended time-course reveals that NHC causes minor reductions in cell viability in HepaRG cells

Cell viability assays were conducted to ensure that any alterations in mitochondrial function observed in functional assays were a result of mitochondrial toxicity being a primary mechanism, and not confounded by secondary toxic effects associated with cell death. Following 5- or 7-days dosing with or without a recovery period, and 14 days dosing without a recovery period, assays to measure ATP and protein content were conducted. Exposure to the positive control chloramphenicol for 28 days resulted in significant decreases in cellular ATP content, but no changes to protein content (Supplementary Fig. S1).

**Fig. 1:**
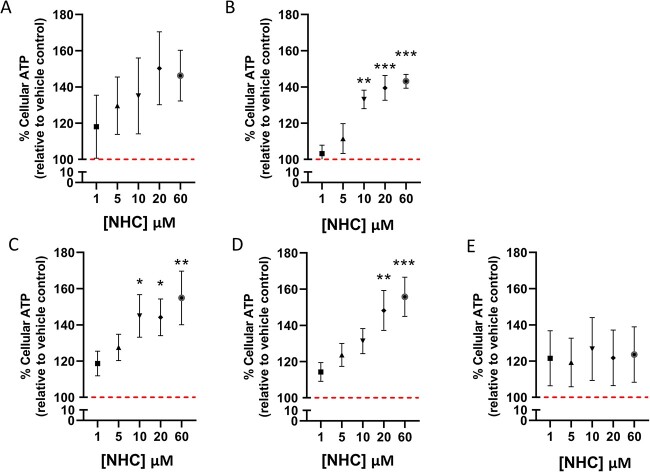
Cellular ATP content of HepaRG cells following prolonged treatment with NHC (0–60 μM). Cellular ATP content (ATP/μg protein) is reported as a percentage of the vehicle control which is marked as a dashed line at 100%. A) Concentration-response following 5 days exposure of HepaRG cells. B) Concentration-response following 7 days exposure of HepaRG cells. C) Concentration-response following 5 days exposure and 7 days recovery of HepaRG cells. D) Concentration-response following 7 days exposure and 7 days recovery of HepaRG cells. E) Concentration-response following 14 days exposure of HepaRG cells. Statistical significance compared to vehicle control was determined using one-way ANOVA followed by Dunnett’s multiple comparisons tests; ^*^p < 0.05, ^*^^*^p < 0.01, ^*^^*^^*^p < 0.001, n = 4, error bars represent standard deviation.

ATP assays did not indicate a significant decrease in cell viability at any timepoint following treatment with 1–60 μM of drug (Fig. 1). When incubated for a maximum of 7 days with or without a 7-day recovery period significant concentration-dependent increases in cellular ATP content were observed in the HepaRG cells, peaking at 155.8 ± 10.8% relative to vehicle control after 7 days of treatment with 60 μM followed by 7 days culture in the absence of NHC (Fig. 1D). This effect appeared to plateau by the final timepoint of 14 days of treatment with NHC, implying that by this timepoint the threshold of HepaRG cells to withstand the deleterious effects of this treatment had been surpassed.

**Fig. 2:**
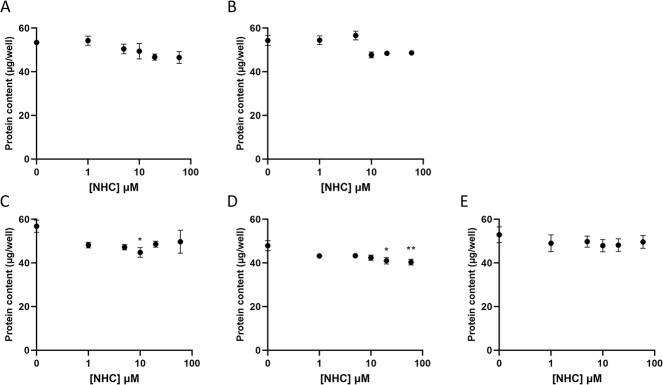
Protein content (μg/well) of HepaRG cells following prolonged treatment with NHC (0–60 μM). A) Concentration-response following 5 days exposure of HepaRG cells. B) Concentration-response following 7 days exposure of HepaRG cells. C) Concentration-response following 5 days exposure and 7 days recovery of HepaRG cells. D) Concentration-response following 7 days exposure and 7 days recovery of HepaRG cells. E) Concentration- response following 14 days exposure of HepaRG cells. Statistical significance compared to vehicle control was determined using one-way ANOVA followed by Dunnett’s multiple comparisons tests; ^*^p < 0.05, ^*^^*^p < 0.01, ^*^^*^^*^p < 0.001, ^*^^*^^*^^*^p < 0.0001, n = 4, error bars represent standard deviation.

Due to the non-proliferative nature of differentiated HepaRG cells, total cellular protein can be used as a marker of cell viability, and thus cytotoxicity. Using this marker, cell viability was found to have significantly decreased following 5 and 7 days of treatment with NHC followed in each case with 7 days of recovery (Fig. 2). This decrease in cell viability was only concentration-dependent after 7 days of treatment and 7 days of recovery (Fig. 2D). When matched for concentration, there was no significant changes to total cellular protein between timepoints.

### NHC has differential effects on mitochondrial DNA copy number in HepaRG cells depending on concentration and timepoint

**Fig. 3:**
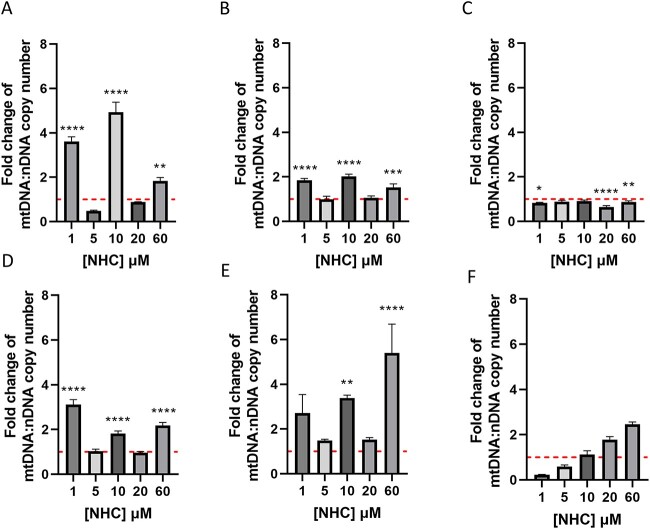
Effect of 3–14 days of NHC treatment (0–60 μM) on mitochondrial DNA copy number. Values represent the ratio of mtDNA/nDNA, and are presented as fold change relative to vehicle. A) Concentration-response following exposure of HepaRG cells NHC for 3 days. B) Concentration-response following 5 days exposure. C) Concentration-response following 7 days exposure. D) Concentration-response following 5 days exposure and 7 days recovery. E) Concentration-response following 7 days exposure and 7 days recovery. F) Concentration-response following 14 days exposure to NHC. Statistical significance compared to vehicle control was determined using one-way ANOVA followed by Dunnett’s multiple comparisons tests; ^*^p < 0.05, ^*^^*^p < 0.01, ^*^^*^^*^p < 0.001, ^*^^*^^*^^*^p < 0.0001, n = 3, error bars represent standard deviation.

A common off-target effect of several nucleoside analogue reverse-transcriptase inhibitors (NRTIs) is the inhibition of mitochondrial DNA polymerase (pol γ).^29^ As mitochondria are essential for physiological cellular function and signalling, it is unsurprising that drug-induced mtDNA depletion has been reported as a key driver of deleterious alterations to bioenergetics, which has led to termination of clinical trials for ribonucleoside analogues in the past.^12^ Exposure to chloramphenicol, a known driver of mitochondrial dysfunction, resulted in time-dependent increases in HepaRG mtDNA copy number (Supplementary Fig. S2A). Following incubation with NHC, mtDNA copy number was generally increased relative to vehicle control (Fig. 3). The most significant increase in mtDNA copy number was observed following 7 days of treatment with 60 μM NHC and 7 days of recovery (5.4 ± 1.3-fold greater than vehicle) (Fig. 3E). Significant decreases to mtDNA copy number were also observed following 7 or 14 days of NHC exposure (Fig. 3C–F). At day 7, mtDNA copy number was consistently lower in NHC-treated cells apart from at 10 μM. Following 14 days of drug exposure, the effect of NHC on mtDNA appeared to be biphasic, with concentrations <10 μM resulting in significantly lower mtDNA copy number (0.3 ± 0.01-fold greater than vehicle at 1 μM), and concentrations >10 μM causing significantly increased mtDNA copy number (2.5 ± 0.1-fold greater than vehicle at 60 μM).

### NHC does not alter expression of mitochondrial proteins in HepaRG cells

**Fig. 4:**
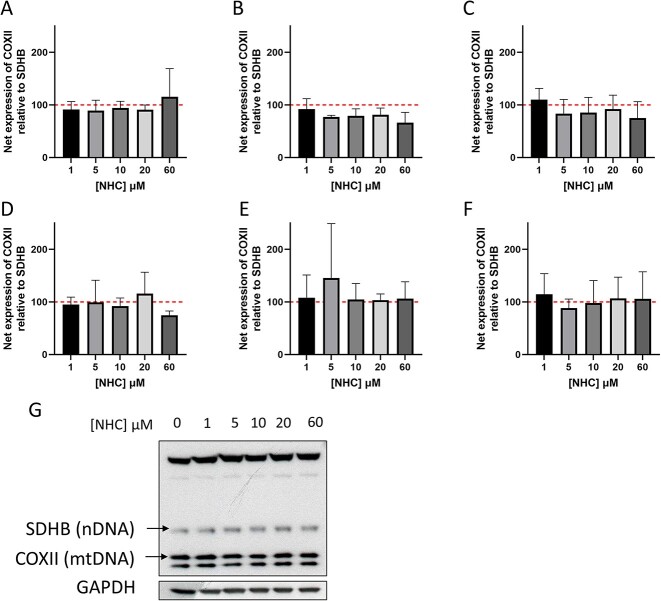
Effect of 3–14 days of NHC treatment (0–60 μM) on the expression ratio of mitochondrial-encoded vs nuclear-encoded mitochondrial proteins. Values represent the ratio of COXII/SDHB expression, and are normalized to the vehicle control which is marked by a dashed line at 100%. A) Concentration-response following exposure of HepaRG cells NHC for 3 days. B) Concentration-response following 5 days exposure. C) Concentration-response following 7 days exposure. D) Concentration-response following 5 days exposure and 7 days recovery. E) Concentration- response following 7 days exposure and 7 days recovery. F) Concentration-response following 14 days exposure to NHC. G) Representative blot from HepaRG cells dosed for 14 days with 0–60 μM NHC. Statistical significance compared to vehicle control was determined using one-way ANOVA followed by Tukey’s multiple comparisons tests; n = 3, error bars represent standard deviation.

As well as the potential to inhibit pol γ, it is well documented that ribonucleoside analogues have the capacity to be incorporated into human mitochondrial RNA (mtRNA) by the mitochondrial DNA-dependent RNA polymerase (POLRMT).^30–32^ As a result of this, ribonucleoside analogues also have the potential to affect the expression of mitochondrial proteins encoded for by mtRNA. In order to investigate the potential of NHC to alter the expression of mtRNA-encoded mitochondrial proteins, the expression of two mitochondrial proteins were compared: a mtRNA-encoded subunit of complex IV, cytochrome-c oxidase 2 (COXII), and the nuclear-encoded iron-sulphur protein subunit of succinate dehydrogenase (SDHB). Unlike the positive control chloramphenicol which induced significant decreases to mtDNA-encoded protein expression relative to nDNA-encoded SDHB (Supplementary Fig. S2B), treatment with NHC did not alter the expression of either nRNA-encoded SDHB or mtRNA-encoded COXII relative to GAPDH (data not shown), or the ratio of SDHB:COXII expression (Fig. 4).

### NHC decreases mitochondrial respiration at supra-clinical concentrations, but does not cause electron transport chain dysfunction

**Fig. 5:**
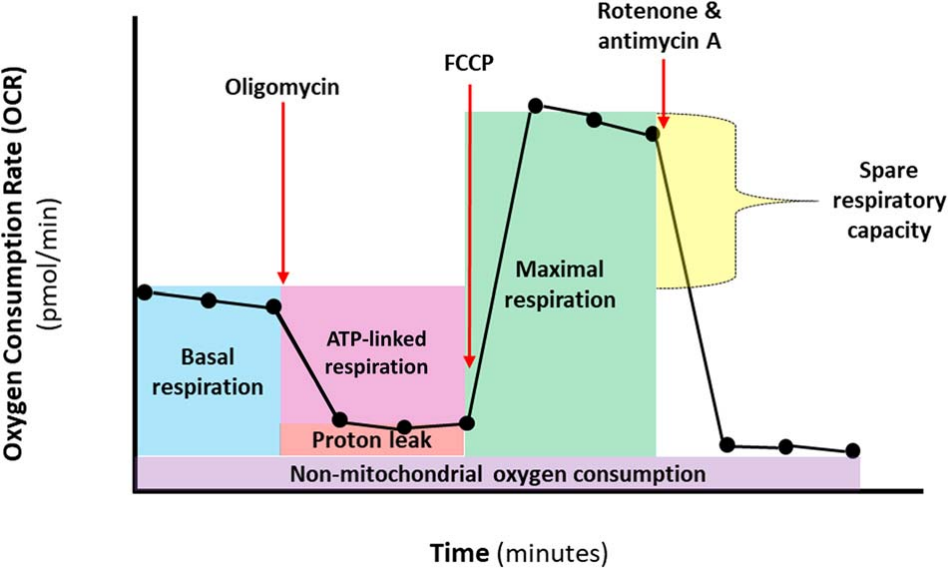
Parameters of mitochondrial respiration measured using the seahorse mitochondrial stress test. The sequence of compound additions is depicted alongside the bioenergetic parameters which can be calculated following this assay.

Mitochondrial respiration was examined by performing mitochondrial stress tests using Seahorse technology. The oxygen consumption rate (OCR) of HepaRG cells was measured following treatment with NHC for 3–14 days to establish a basal respiration (BR) rate, and after cells were subjected to the addition of the ATP synthase inhibitor oligomycin, mitochondrial electron transport chain (ETC) uncoupler FCCP, and inhibitors of complexes I and III respectively, rotenone/antimycin A. Sequential addition of these pharmacological inhibitors of oxidative phosphorylation (OXPHOS) (Fig. 5) enables the amount of oxygen consumed for several bioenergetic parameters to be established (Table 4). Exposure to chloramphenicol for 28 days resulted in significant reductions in multiple bioenergetic parameters in HepaRG cells, consistent with ETC dysfunction (Supplementary Fig. S3).

**Table 4:** Formulas for mitochondrial stress test respiratory parameters defined by Agilent Technologies.

Parameter	Formula
Basal respiration	(Last measurement before first oligomycin injection) − (non-mitochondrial oxygen consumption rate)
Maximal respiration	(Maximum rate measurement following FCCP injection) − (non-mitochondrial oxygen consumption)
ATP-linked respiration	(Last rate measurement prior to oligomycin injection) − (minimum rate measurement after oligomycin injection)
Spare respiratory capacity	(Maximum respiration) − (basal respiration)
Spare respiratory capacity (%)	(Maximal respiration)/(basal respiration) × 100
Coupling efficiency (%)	(ATP-linked respiration)/(basal respiration) × 100
Proton leak	(Minimum rate measured following oligomycin injection) − (non-mitochondrial oxygen consumption)
Non-mitochondrial oxygen consumption	Minimum respiration rate following rotenone/antimycin A injection

**Fig. 6:**
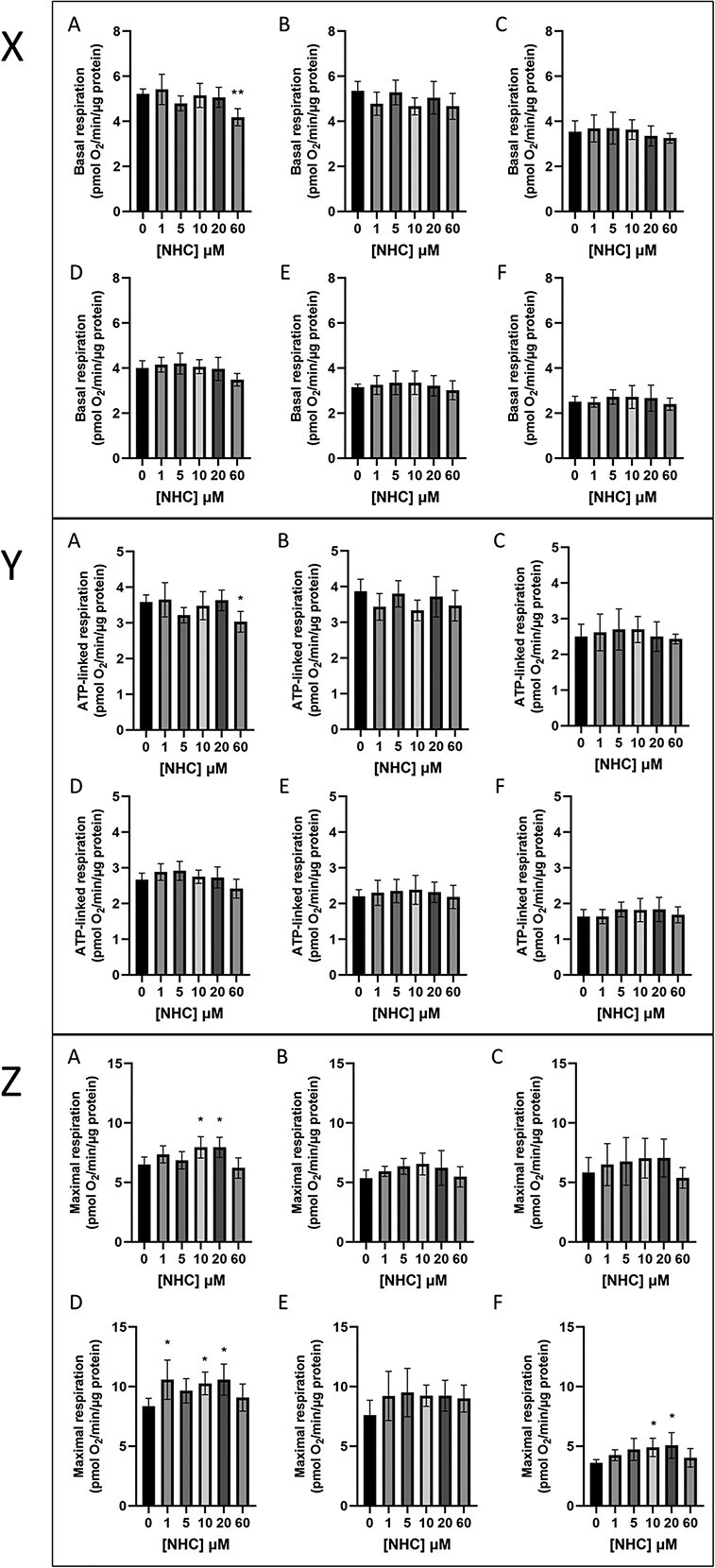
Effect of 3–14 days NHC treatment (0–60 μM) on the basal, maximal, and ATP-linked respiratory activity of HepaRG cells. X: Basal respiration. Y: ATP-linked respiration. Z: Maximal respiration. For each panel treatment duration was as follows: A) 3 days of NHC treatment; B) 5 days of NHC treatment; C) 7 days of NHC treatment; D) 5 days of NHC treatment followed by 7 days of recovery; E) 7 days of NHC treatment followed by 7 days of recovery; F) 14 days of NHC treatment. Statistical significance compared to vehicle control was determined using one-way ANOVA followed by Dunnett’s multiple comparisons tests; ^*^p < 0.05, ^*^^*^p < 0.01, n = 6, error bars represent standard deviation.

After three days of treatment with supra-clinical concentrations of NHC (60 μM), BR of HepaRG cells was significantly reduced by almost 20% relative to vehicle, from 5.2 ± 0.2 to 4.2 ± 0.4 pmol O_2_/min/μg protein (p = 0.0021) (Fig. 6X). This decrease in BR was also reflected by a 15% decrease in ATP-linked respiration (ALR), which again was only shown to be significant following 3 days of treatment with 60 μM NHC (Fig. 6Y). No other NHC-induced significant changes to BR or ALR were observed at any timepoint. The maximal respiratory capacity of HepaRG cells was not significantly decreased by NHC treatment at any times or concentrations tested (Fig. 6Z). However, both BR and ALR were found to decrease significantly with time; in vehicle-treated cells, BR and ALR decreased by over 50% to 2.5 ± 0.3 and 1.6 ± 0.2 pmol O_2_/min/μg protein respectively between day 3 and day 14. This time-dependent decrease was consistent across all concentrations of NHC tested.

Maximal respiration (MR) was significantly increased in HepaRG cells treated with 10 or 20 μM NHC for 3 or 14 days, and 1, 10, or 20 μM NHC for 5 days with a 7-day recovery period when compared to respective vehicle control, peaking at over 1.4-fold greater than vehicle at 5.1 ± 1.1 pmol O_2_/min/μg protein following 14 days of treatment with 20 μM NHC (Fig. 6Z). MR rates of HepaRG cells treated with 60 μM NHC across all timepoints tested were not significantly lower than those of cells treated with 1–20 μM drug when matched for length of drug exposure. Unlike BR and ALR, the relationship between NHC exposure length and MR was non-monotonic. The inclusion of a 7-day recovery period was found to significantly increase MR when compared to any other timepoint and matched for concentration (p < 0.05). This increase was consistently reversed by day 14 of consistent NHC exposure, where for all vehicle and NHC-treated cells excluding those at 5 μM, MR was significantly lower when compared with day 3 (p < 0.01).

**Fig. 7:**
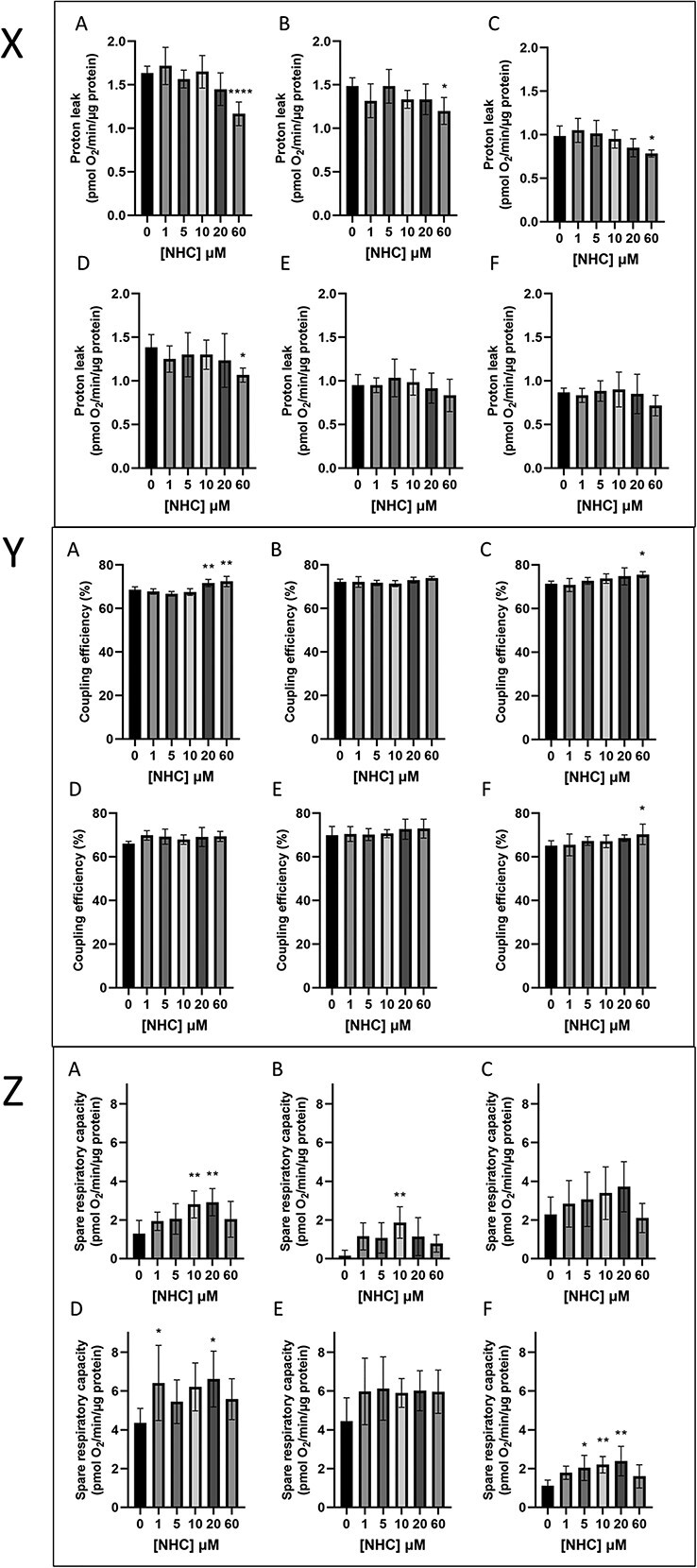
Effect of 3–14 days NHC treatment (0–60 μM) on the proton leak, coupling efficiency, and spare respiratory capacity of HepaRG cells. X: Proton leak. Y: Coupling efficiency (%). Z: Spare respiratory capacity. For each panel treatment duration was as follows. A) 3 days of NHC treatment; B) 5 days of NHC treatment; C) 7 days of NHC treatment; D) 5 days of NHC treatment followed by 7 days of recovery; E) 7 days of NHC treatment followed by 7 days of recovery; F) 14 days of NHC treatment. Statistical significance compared to vehicle control was determined using one-way ANOVA followed by Dunnett’s multiple comparisons tests; ^*^p < 0.05, ^*^^*^p < 0.01, ^*^^*^^*^p < 0.001, ^*^^*^^*^^*^p < 0.0001, n = 6, error bars represent standard deviation.

Reduction in BR following treatment with 60 μM NHC was accompanied by a reduction in proton leak (PL), dropping by 25% from 1.6 ± 0.1 to 1.2 ± 0.1 pmol O_2_/min/μg protein after 3 days (p < 0.0001) (Fig. 7X). This significant decrease was observed at days 3, 5 (with and without recovery period), and 7 of 60 μM NHC treatment, after which point the decrease in PL of HepaRG treated with this concentration of the drug did not breach the threshold of significance relative to vehicle control. Modest concentration-dependent increases in percentage coupling efficiency (ce) were also implied at all timepoints, but significant only at days 3, 7, and 14. The most significant increases in PL were observed at day 3 following exposure to 20 and 60 μM NHC, with ce increasing from 68.6 ± 1.4% to 71.7 ± 1.7% and 72.4 ± 2.3% respectively (Fig. 7Y). This is likely a result of the concurrent reductions in PL. Interestingly, treatment with NHC also revealed non-monotonic alterations in spare respiratory capacity (SRC) observed within HepaRG cells (Fig. 7Z). When HepaRG cells were exposed to NHC without a recovery period, SRC appeared to increase in a concentration-dependent manner from 1–20 μM, before then beginning to decrease at the 60 μM concentration. The most significant concentration-dependent increases in SRC were observed after 14 days of NHC treatment, implying a time-dependence as well as the evident concentration-dependence. At this timepoint, significant increases in SRC were elicited by 5, 10, and 20 μM NHC, with 20 μM causing SRC to double to 2.4 ± 0.8 pmol O_2_/min/μg protein. Timepoints which included a 7-day recovery period did not reflect this concentration-dependent pattern, but resulted in increased SRC compared to timepoints lacking a recovery period.

## Discussion

Nucleoside analogues have been instrumental in the effective management of viruses such as HIV and hepatitis, and more recently have become invaluable tools for treating SARS-CoV-2 since the emergence of the virus in late 2019 thanks to their ability to prevent the successful replication of viral DNA or RNA.^33–35^ However, the ability to intercept viral polymerases including RdRp is also a key driver of toxicity for some nucleoside analogues that lack specificity to the viral genome. The mitochondrial genome is especially susceptible to nucleoside analogue-induced toxicity due to the lack of adequate intrinsic proofreading and repair mechanisms; human POLRMT in particular has a high intrinsic error rate and has been shown to incorporate many ribonucleoside analogues.^30^^,^^31^^,^^36^^,^^37^ Due to the ubiquitous nature of mitochondria within human cells and the essential role they play in driving and maintaining physiological cellular functions, disruption to these organelles by nucleoside analogues can be catastrophic, as was infamously the case with fialuridine.^19^^,^^20^ At the time of writing there are no published reports of clinical mitochondrial toxicity being induced by either molnupiravir or its active metabolite NHC. However, POLRMT has been shown to be capable of incorporating NHC at a low efficiency in vitro, which could be a cause for concern due to the pivotal role POLRMT has in the regulation of mammalian mtDNA replication.^32^^,^^38^ Reassuringly, despite the ability to incorporate NHC into mtRNA, Sticher et al. found that NHC does not appear to substantially impair mitochondrial function in HepG2 cells following 14 days of treatment.^32^ HepG2 have however previously been shown to be limited in their ability to detect nucleoside analogue-induced mitochondrial dysfunction, so confirming these findings in HepaRG cells would greatly strengthen confidence in their translatability.^20^

A decrease in total cellular protein content, indicative of a reduction in HepaRG viability, was found to be significant following 5 and 7 days of NHC treatment followed by a 7-day recovery period. The fact that significant reductions in protein content were observed only when NHC treatment was followed by a drug-free period may be a result of vehicle-treated cells being able to recover more effectively in this recovery period. Using ATP assays to further examine cell viability, we found that NHC did not cause significant reductions in ATP indicative of cytotoxicity in HepaRG cells at any timepoint tested. In fact, when cells were dosed for a maximum of 7 days, significant increases in ATP levels were observed. One potential explanation for this is that by hindering RNA-dependent translation and transcription within the mitochondria, treatment with NHC may lead to ATP surplus within cells.^39^ However, no significant decreases were seen in the expression of mtDNA-encoded proteins within treated HepaRG cells, suggesting that this may not be the primary explanation of an ATP increase in this case. Another possible explanation could be that this is a compensatory effect within the cells in order to withstand treatment with NHC. The absence of this concentration-dependent ATP increase at day 14 implies that the temporal threshold of the cells to tolerate treatment may have been breached.

An IC50 was not established in HepaRG cells, meaning it is greater than 60 μM and therefore higher than reported previously in HepG2 cells.^15^^,^^32^ HepaRG cells are known to express human equilibrative nucleoside transporter 1 (hENT1) at considerably lower levels than PHH, with proteomic analysis revealing a significant fold difference in relative expression of −9.17.^20^ This is in contrast to HepG2 cells where hENT1 expression did not significantly differ to that of PHH (fold change 1.89, p > 0.05).^20^ Whilst this study by Jolly et al. may not be entirely representative of physiological hENT1 expression due to the small sample size of 3 PHH donors, it highlights the disparity in hENT1 expression between HepaRG and HepG2. As is the case for several nucleoside analogues, hENT1 has been reported to be key in the cellular uptake of NHC.^40^ Therefore, the relative under-expression of hENT1 in HepaRG cells is likely to make them less susceptible to NHC toxicity than HepG2 cells. Unlike uptake, metabolism of NHC is unlikely to contribute to variation in tissue or cell-line specific susceptibility as conversion to the active triphosphate form is efficient and ubiquitous, although the identities of the host kinases responsible for the phosphorylation of NHC are as yet unknown.^41^^,^^42^ Furthermore, differentiated HepaRG cells are non-proliferative meaning lack of proliferation cannot be interpreted as cytotoxicity when using this in vitro model, which may influence IC50 readouts depending on the assay conducted.

NHC was found to induce non-monotonic alterations to mtDNA copy number. Significant increases in copy number compared to vehicle were observed at days 3 and 5 in cells treated with 1, 10, or 60 μM NHC, but by day 7 mtDNA was found to be consistently decreased relative to vehicle control. In cells treated for 5 or 7 days followed by a 7-day drug-free recovery period, substantial significant increases were again observed following exposure to 1, 10, or 60 μM NHC. Increases in mtDNA copy number as a result of compensatory mechanisms are well documented within the literature following both drug insult and disease onset, and also occurred following incubation with the positive control chloramphenicol.^43–46^ The temporal pattern of the observed fluctuations in mtDNA copy number are indicative of a compensatory response, with cells potentially overcompensating at days 3 and 5 until they can no longer do so following 7 days of consecutive NHC exposure. Upon the inclusion of a recovery period, it appears that HepaRG cells have the capacity to again increase mtDNA potentially in an attempt to protect themselves against NHC-induced toxicity. At day 14, mtDNA displays a biphasic response to NHC treatment, with concentrations <10 μM resulting in decreased mtDNA copy numbers, and concentrations >10 μM eliciting an increase. Further work would be needed to confirm the exact cause of this biphasic response, but it is likely a result of compensatory upregulation of mitochondrial biogenesis as a result of cell stress.

No significant alterations in mitochondrial protein expression were observed following NHC exposure at any concentrations or timepoints tested. Previous studies have reported conflicting effects on these parameters, with NHC being shown to induce significant increases in mtRNA in one study, yet decrease mitochondrial protein expression in another.^15^^,^^32^ The reported significant decrease in protein expression was observed following prolonged (14 days) of treatment in HepG2 cells, so differences in susceptibility here could again be at least partially explained by the relatively low hENT1 expression of HepaRG cells relative to HepG2. The proliferative phenotype of HepG2 vs the differentiated HepaRG is also likely to increase their susceptibility to NHC-induced toxicity, as has been observed in vitro in various models.^47^^,^^48^ Proliferative cells usually consume greater amounts of energy in order to fulfil their biosynthesis needs, resulting in depleted ATP reserves and thus less flexibility to adapt to external stress.^48^ hENT1 expression of proliferative cancer cell lines is also reported to be closely linked to cell cycle, almost doubling between G1 and G2/M phases.^49–51^ Considering this, it is possible that HepG2 cells are capable of NHC uptake beyond clinically relevant levels. Additionally, there may be target-specific discrepancies in NHC effect contributing to the conflicting findings.

Respirometry was used to investigate the potential of NHC to perturb cellular bioenergetics via the mitochondrial ETC. Treatment with NHC revealed a complex and interesting bioenergetic profile. BR was decreased only at the highest concentration (60 μM), and this was only significant at the earliest timepoint tested (day 3). This decrease was accompanied by a significant reduction in PL, which likely accounts for the observed decrease in BR as well as the modest concentration-dependent increases in ce. Non-monotonic alterations in SRC were observed across almost all timepoints, with SRC showing concentration-dependent increases between 1–20 μM, then beginning to decline at 60 μM. Though further experiments would be required to confirm this, these changes imply an attempt at a protective response by the mitochondria until a threshold is reached between 20–60 μM. For example, increased mitochondrial fusion may occur in response to external insult, resulting in the extension of mitochondrial networks and subsequent increases in SRC and ATP synthesis.^52–54^ This hypothesis would support the observed increases in SRC and ATP content, as although SRC peaks at 20 μM NHC, it is possible that ATP content remains elevated at 60 μM due to compensatory elevations in ATP reserves. The reported Cmax of NHC in clinical trials is around 16 μM, which means that that the compensatory mechanisms of the mitochondria occur at clinically relevant concentrations.^24^ Interestingly, when treatment included a 7-day recovery period, SRC did not follow the same concentration-dependent pattern, and after a 7-day treatment followed by a 7-day recovery the cells treated with 60 μM NHC had the highest SRC relative to vehicle than any other concentrations. It has previously been reported that HepaRG cells are capable of overcoming treatment-induced damage when allowed a “recovery period” of maintenance in standard culture medium following cessation of treatment.^55^ SRC is a parameter which is highly influenced by mitochondrial homeostasis, and as a result can be influenced heavily by external stimuli.^56^ It has previously been reported that an increase in mitochondrial biogenesis can lead to increased SRC.^57^ The increases in mtDNA observed following NHC treatment in HepaRG cells could at least partially account for the elevations in SRC. Another likely explanation is that in order to maintain the plasticity essential to respond to future stresses, exerting stress on mitochondria via external stimuli such as drugs can in fact lead to compensatory increases in SRC.^56^^,^^58^ In addition to this, HepaRG have intrinsically higher SRC than less organotypic cancer cell lines such as HepG2, potentially due to the increased energy expenditure of the highly proliferative HepG2.^58^ This could explain why the same initial increases in SRC are not observed following NHC treatment in HepG2 cells, as they have significantly lower basal SRC combined with a more glycolytic bioenergetic phenotype than HepaRG cells, thus making them more susceptible to acute cellular stress.^58–60^

Although a decrease in PL is frequently regarded as purely being an indicator of good mitochondrial membrane and ETC integrity, this does not tell the whole story. Induction or reduction of PL is a complex and multifactorial occurrence which plays central roles in mitochondrial processes including the generation of reactive oxygen species (ROS) and the organelles response to inflammatory stress.^61^ Although there are conflicting reports within the literature, it is now widely accepted that an increase in uncoupling leads to decreased mitochondrial ROS generation, and thus plays an important protective role.^61–63^ Therefore, it is possible that the significant reductions in PL observed after treatment with 60 μM NHC are not an indicator of improved ETC integrity, but rather the deleterious result of the mitochondria no longer being able to induce PL as a protective mechanism. This conclusion is supported by the fact that SRC also begins to decrease at 60 μM NHC compared to the lower concentrations tested, again implying that the threshold for sufficient protective mechanisms initiated by the mitochondria has been breached.

As well as concentration-dependent effects, there were clear temporal effects on OCR. These differences were evident even in the untreated cells, with BR, MR, ALR, and PL being significantly reduced at day 14 compared to day 3 in vehicle-exposed HepaRG. As is the case with all cell lines, functional decline is a consequence of cellular aging, and as such HepaRG are only used up to passage 20 in accordance with supplier guidance. Time-dependent decreases in cellular and mitochondrial oxygen consumption in HepaRG cells has been reported previously, and importantly do not seem to impact the long-term (28 days post-differentiation) stability of HepaRG function, including cytochrome P450 expression and activity.^64^^,^^65^ Additionally, long-term culture did not appear to affect the utility of HepaRG cells in toxicological studies.^65^

## Conclusion

Overall, this work indicates that NHC, the active metabolite of the COVID-19 prodrug molnupiravir, does not cause mitochondrial toxicity beyond the threshold of tolerability in HepaRG cells at clinically relevant exposures, but may cause minor perturbations to physiological cell function. Despite sub-physiological expression of hENT1, HepaRG remain a more representative model of human liver than HepG2 cells and allow for the recapitulation of clinically relevant drug exposure periods; therefore, these findings provide additional assurance of the mitochondrial safety of NHC. No significant reductions in cellular ATP content or mtDNA-encoded proteins were detected, however fluctuations in mtDNA copy number were evident, indicative of a compensatory response to cellular stress. This stress may be intra- or extra-mitochondrial, and did not result in mitochondrial toxicity when defined as a decrease in mitochondrial function indicated by the included endpoints. Additionally, minor alterations in cellular bioenergetic phenotype were observed that given the propensity of nucleoside analogues to interfere with transcription and translation of the mitochondrial genome may warrant further investigation, particularly if the dosing regimen of molnupiravir is ever extended beyond the current 5 days indicated for the treatment of COVID-19.^21^ The ability of mitochondria to withstand toxicity up to a critical value has been termed the “threshold effect,” and is well documented; for example, it has been reported that 80% inhibition of complex III is required before ATP synthesis is significantly compromised.^20^^,^^32^^,^^66–68^ Phenomena such as this mean that the consideration of delayed onset of mitochondrial toxicity is vital, and will remain so as novel nucleoside analogues are developed in the future.

## Supplementary Material

Supp_Fig_1_tfae012

Supp_Fig_2_tfae012

Supp_Fig_3_tfae012

Supplementary_information_(1)_tfae012
