# Supplementary figures and images for: Assessing the mitochondrial safety profile of the molnupiravir active metabolite, β-d-N4-hydroxycytidine (NHC), in the physiologically relevant HepaRG model
Source: Toxicol Res (Camb). 2024 Feb 7;13(1):tfae012. doi: 10.1093/toxres/tfae012 (PMC10848230; doi:10.1093/toxres/tfae012)

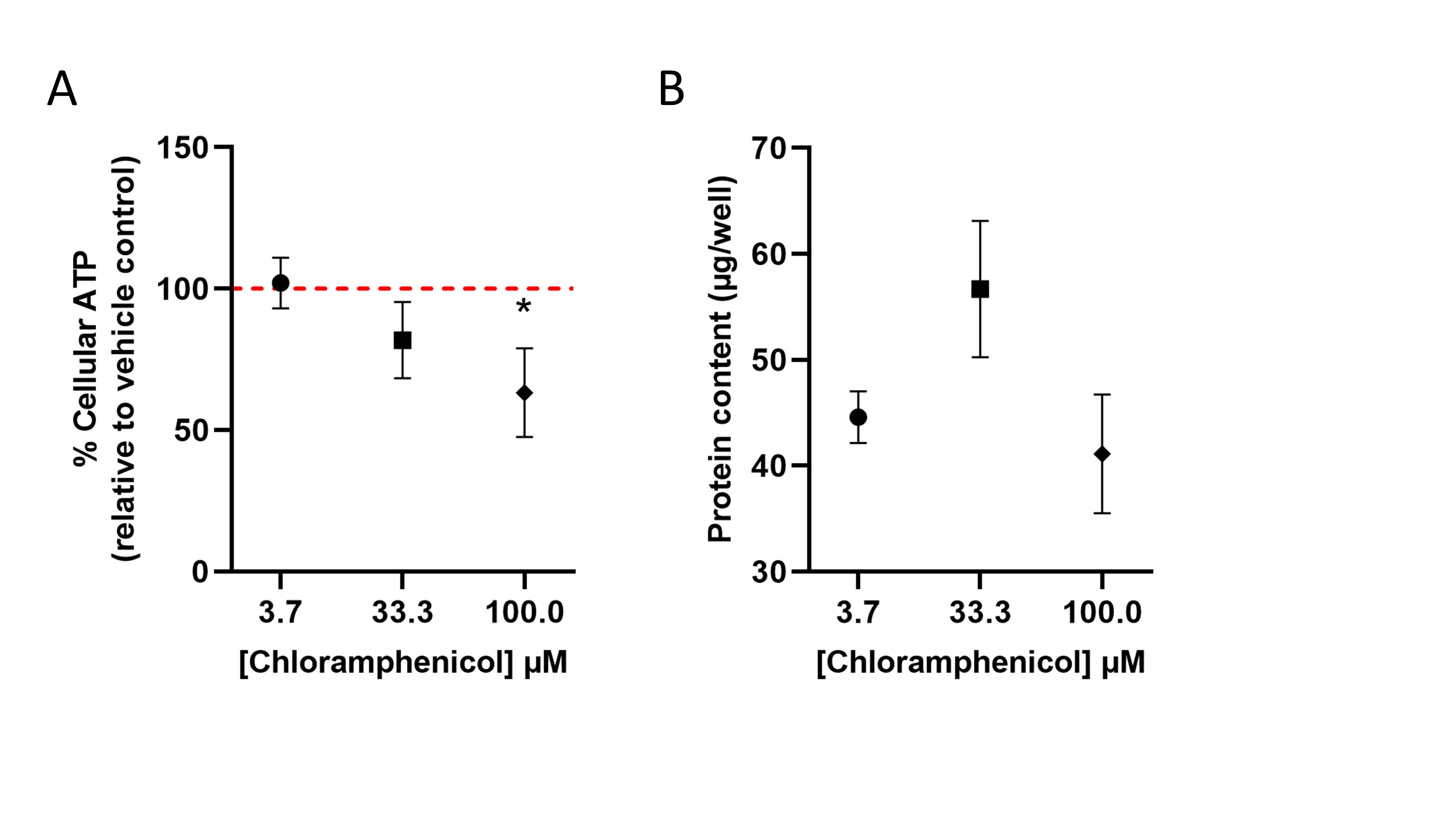

Supplement: Supp_Fig_1_tfae012 [file supp_fig_1_tfae012.jpeg]

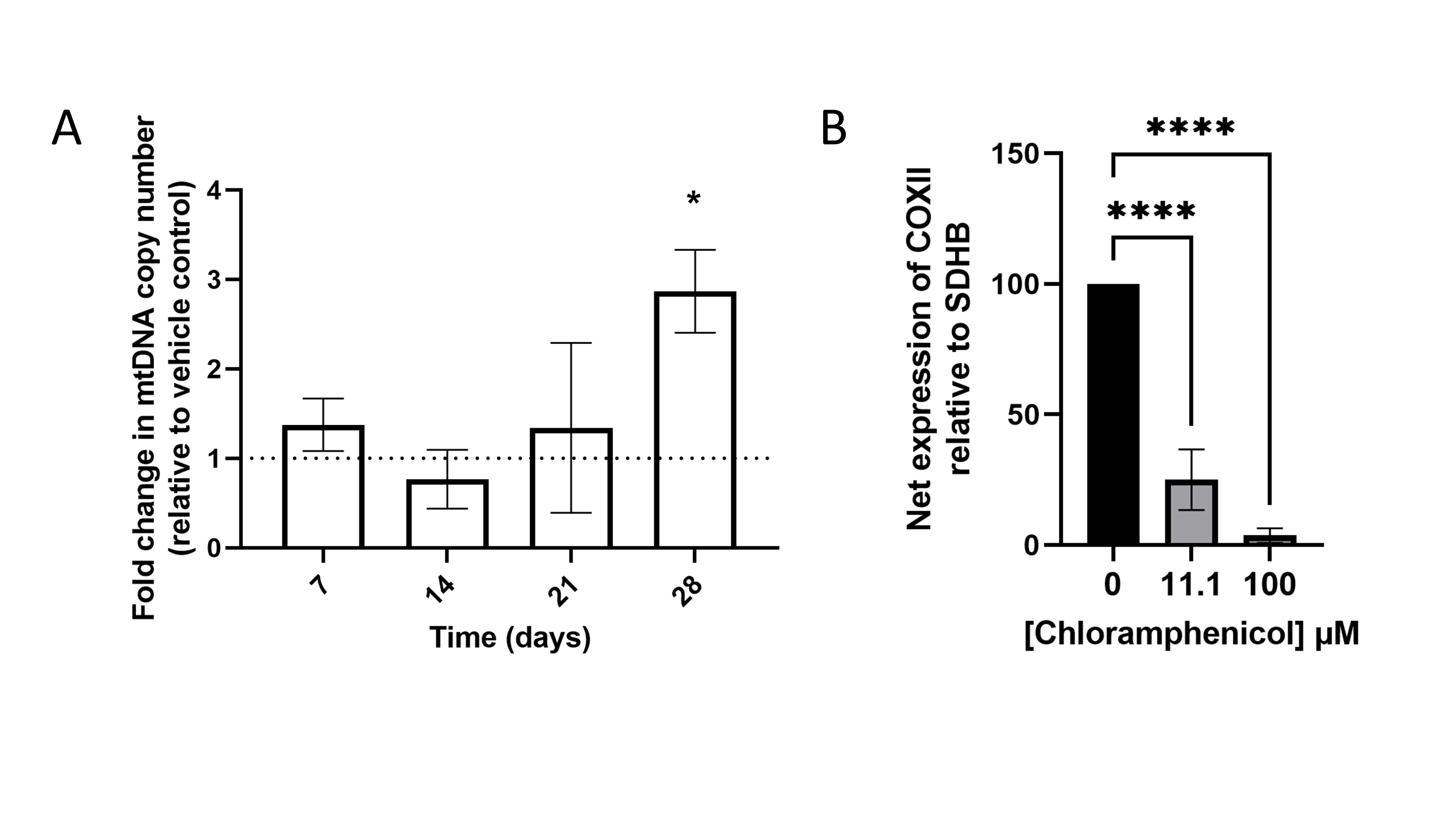

Supplement: Supp_Fig_2_tfae012 [file supp_fig_2_tfae012.jpeg]

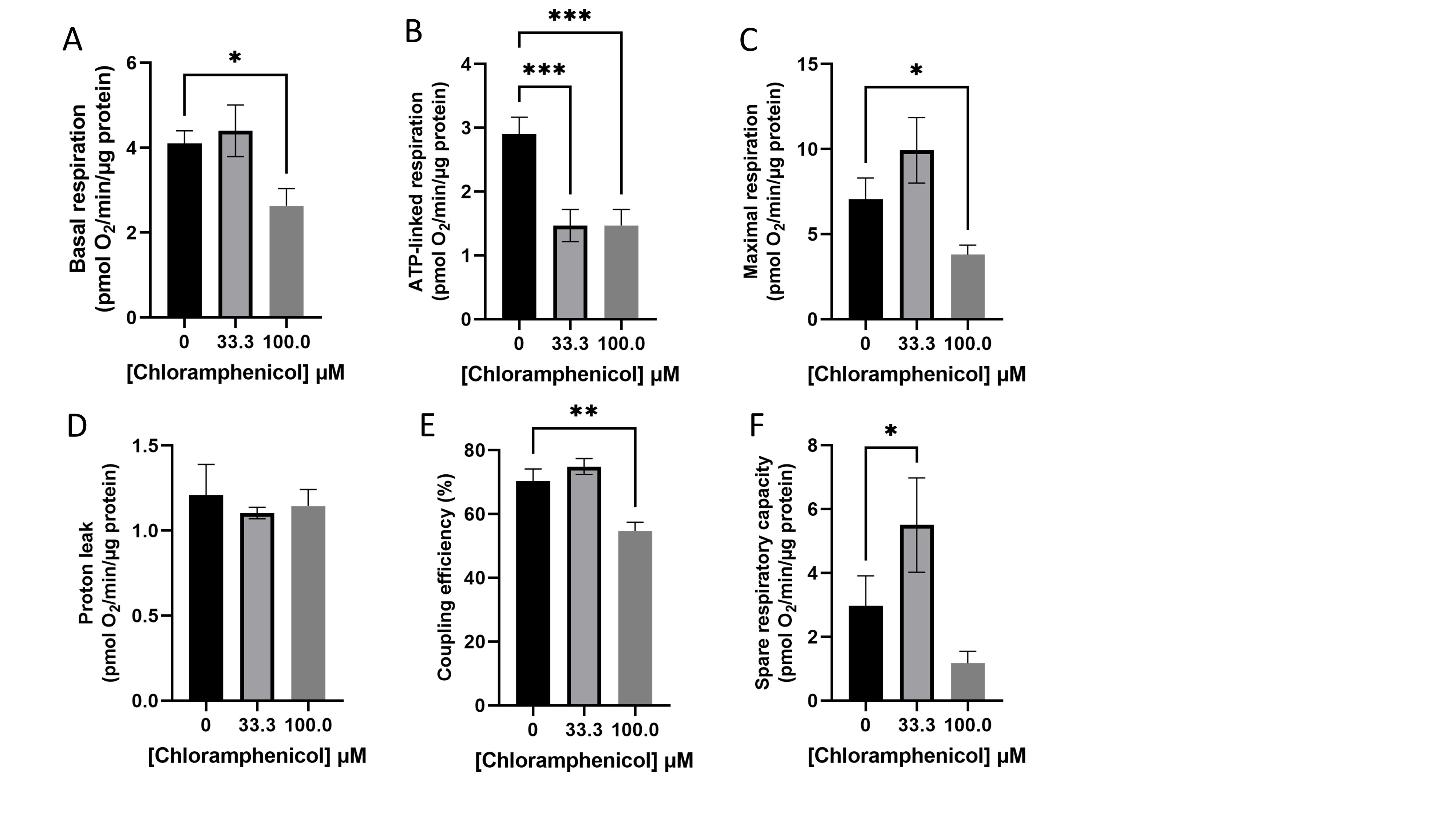

Supplement: Supp_Fig_3_tfae012 [file supp_fig_3_tfae012.jpeg]
