# Supplementary material for: Assessing the mitochondrial safety profile of the molnupiravir active metabolite, β-d-N4-hydroxycytidine (NHC), in the physiologically relevant HepaRG model
Source: Toxicol Res (Camb). 2024 Feb 7;13(1):tfae012. doi: 10.1093/toxres/tfae012 (PMC10848230; doi:10.1093/toxres/tfae012)
Supplement: Supplementary_information_(1)_tfae012 [file supplementary_information_(1)_tfae012.docx]

**Figure S1. Cellular ATP and protein content of HepaRG cells following 28 days of exposure to chloramphenicol (0 – 100.0 µM).** A: cellular ATP content (ATP/μg protein) following 28 days of exposure to chloramphenicol is reported as a percentage of the vehicle control which is marked as a dashed line at 100 %. B: protein content (µg/well). Statistical significance compared to vehicle control was determined using one-way ANOVA followed by Dunnett’s multiple comparisons tests; * p < 0.05, n = 3, error bars represent standard deviation.

**Figure S2. Effect of chloramphenicol treatment on mitochondrial DNA copy number and the expression of mitochondrial-encoded proteins**. A: Values represent the ratio of mtDNA/nDNA, and are presented as fold change relative to vehicle following exposure to 11.1 µM chloramphenicol for 7 – 28 days. B: values represent the ratio of COXII/SDHB expression following 28 days of exposure to chloramphenicol, and are normalised to the vehicle control. Statistical significance compared to vehicle control was determined using one-way ANOVA followed by Dunnett’s multiple comparisons tests; * p < 0.05, ** p < 0.01, *** p < 0.001, **** p < 0.0001, n = 3, error bars represent standard deviation.

**Figure S3. Effect of 28 days of chloramphenicol exposure (0 – 100.0 µM) on mitochondrial bioenergetics within HepaRG cells.** A: Basal respiration. B: ATP-linked respiration. C: Maximal respiration. D: Proton leak. E: Coupling efficiency (%). F: Spare respiratory capacity. Statistical significance compared to vehicle control was determined using one-way ANOVA followed by Dunnett’s multiple comparisons tests; * p < 0.05, ** p < 0.01, *** p < 0.001, n = 3, error bars represent standard deviation.
